# Engineering quantum states from a spatially structured quantum eraser

**DOI:** 10.1126/sciadv.adm9278

**Published:** 2024-07-24

**Authors:** Carlo Schiano, Bereneice Sephton, Roberto Aiello, Francesco Graffitti, Nijil Lal, Andrea Chiuri, Simone Santoro, Luigi Santamaria Amato, Lorenzo Marrucci, Corrado de Lisio, Vincenzo D’Ambrosio

**Affiliations:** ^1^ Dipartimento di Fisica, Università di Napoli Federico II, Complesso Universitario di Monte S. Angelo, Via Cintia, 80126 Napoli, Italy.; ^2^ Institute of Photonics and Quantum Sciences, School of Engineering and Physical Sciences, Heriot-Watt University, Edinburgh EH14 4AS, UK.; ^3^ Enea–Centro Ricerche Frascati, via E. Fermi 45, 00044 Frascati, Italy.; ^4^ Italian Space Agency (ASI), Centro di Geodesia Spaziale ‘Giuseppe Colombo’, Località Terlecchia, 75100 Matera, Italy.; ^5^ CNR-ISASI, Institute of Applied Science and Intelligent Systems, Via Campi Flegrei 34, 80078 Pozzuoli (NA), Italy.

## Abstract

Quantum interference is a central resource in many quantum-enhanced tasks, from computation to communication. While usually occurring between identical photons, it can also be enabled by performing projective measurements that render the photons indistinguishable, a process known as quantum erasing. Structured light forms another hallmark of photonics, achieved by manipulating the degrees of freedom of light, and enables a multitude of applications in both classical and quantum regimes. By combining these ideas, we design and experimentally demonstrate a simple and robust scheme that tailors quantum interference to engineer photonic states with spatially structured coalescence along the transverse profile, a type of quantum mode with no classical counterpart. To achieve this, we locally tune the distinguishability of a photon pair by spatially structuring the polarization and creating a structured quantum eraser. We believe that these spatially engineered multiphoton quantum states may be of significance in fields such as quantum metrology, microscopy, and communication.

## Teaser

Quantum correlations can be shaped by combining structured light with quantum erasing.

## INTRODUCTION

In the Hong-Ou-Mandel (HOM) effect, quantum interference occurs when two indistinguishable photons, entering a beamsplitter (BS), take the same output path (photon bunching) (*1*). Although this effect is typically investigated by tuning the temporal matching between two identical photons, the indistinguishability required for the interference to happen makes it fundamentally responsive to any change of the photon’s degrees of freedom, from polarization (*2*, *3*) to frequency (*4*, *5*) and time bins (*6*) as well as for collinear spatial modes (*7*), including multiparticle (*8*, *9*) and high-dimensional scenarios (*10*–*13*). For this reason, the effect can be used as a versatile tool to measure a wide array of variations between the two inputs, enabling quantum-enhanced measurements (*3*, *14*, *15*), which have been extended to the spatial degree of freedom upon the advent of single-photon cameras (*16*–*20*). The fundamentally quantum nature of HOM interference also lends itself to tests of quantum mechanics (*21*, *22*), among which a notable example is the quantum eraser (*2*, *23*). Such a paradigm allows one to restore quantum interference, even if two photons are made distinguishable before entering the BS. This is achieved by projecting the outputs of both exit ports onto a basis that cannot yield information on which path the photon took through the BS and so making the photons effectively indistinguishable. Moreover, such a projection process allows one to edit the state by tuning photon bunching/antibunching behavior, thus lending the quantum eraser to be used as a structuring tool.

The ability to tailor the structure of light forms a very powerful concept in modern optics, spanning both the classical and the quantum regime (*24*). For instance, in the classical realm, it exhibits enhanced sensing (*25*–*29*), microscopy (*30*), and communication (*31*) capabilities, while in the quantum one, it provides a test bed for quantum mechanics (*32*, *33*), secure high-dimensional communication (*34*), and increased resilience to noise (*35*–*37*). Accordingly, there is a strong interest in tailoring complex or new structures, such as designing across nonlocal degrees of freedom for quantum skyrmions (*38*) or harnessing inhomogeneity for complex entanglement structures (*39*–*41*). All degrees of freedom addressed so far in the context of structured light, even in quantum regime, are related to classical light (*42*). In this work, we introduce a scheme, based on quantum interference, to engineer states that are instead directly structured in a quantum feature of the electromagnetic field (i.e., photonic coalescence) and thus provide a type of structured quantum light without a classical counterpart. This way, we can combine the advantages of both concepts, holding the possibility for direct impact in applications based on quantum interference or structured light as well as unlocking new prospects.

Quantum interference is typically exploited in standard photon number engineering, where bunching forms NOON states (*43*–*45*) and, similarly, antibunching provides nonlocal entangled states (*46*). Moreover, coincidence detection acts as a filter for particular Bell and high-dimensional spatial states (*10*), while altering the modal distinguishability between photons facilitates space-time entanglement engineering (*47*, *48*) or the population of spatial modes (*7*, *12*). Such state engineering can be extended to photon subtraction or additions with continuous variables (*49*). While, in these cases, interference was used to postselect or allocate photons to particular spatial modes, here, we demonstrate a scheme to spatially structure the quantum erasing process itself, thus obtaining a photonic state that is directly structured in photonic coalescence. To do this, we harness the innate dependency of the HOM effect on identical conditions to demonstrate how spatially tailoring a degree of freedom can allow one to spatially tailor quantum interference. We achieve this by exploiting geometric phase devices to generate a nonuniform spatial structure in the polarization degree of freedom and, having done so, a spatially varying structure in the distinguishability for the input photons. We therefore implement a quantum eraser (*2*) via polarization projective measurements to study the caveats associated with these engineered states and elicit conditions where coalescent structures can be heralded or suppressed. In particular, we demonstrate bunching and antibunching distribution engineering across the transverse mode profile by exploiting the simple generation of vector vortex (VV) modes. This approach, however, may be generalized to achieve arbitrary freedom in engineering this fundamentally quantum property toward developing a unique and versatile tool for applications in fields such as quantum metrology, microscopy, and communication.

## RESULTS

### Concept

**Fig. 1.**
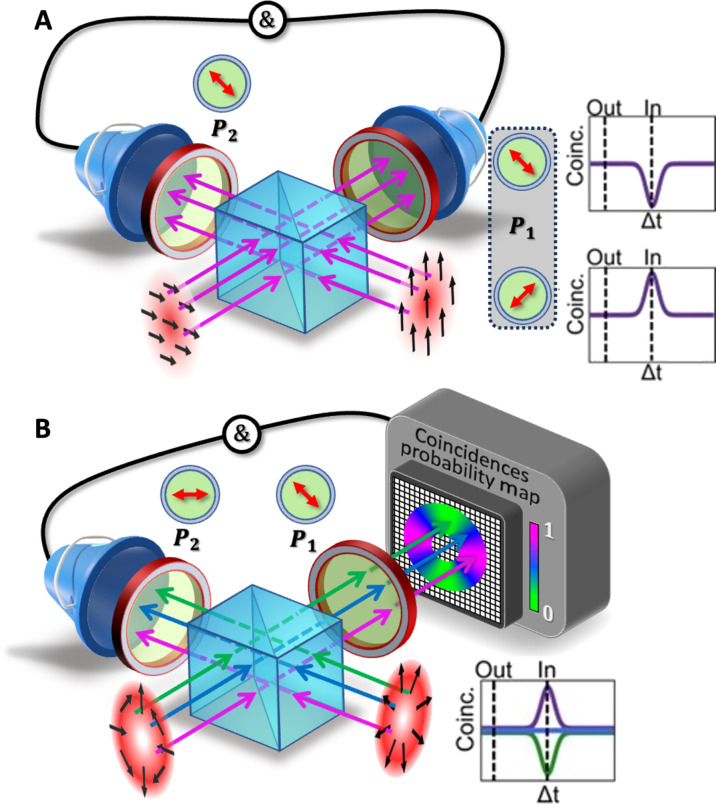
Spatially engineering quantum interference. (**A**) In a quantum eraser, two photons in different polarization states are quantum interfering when their which-path information is erased through two polarizers. Depending on the polarizer orientation, it is possible to tune photon coalescence from bunching to antibunching. This is reflected in a HOM curve (when using bucket detection of coincidences) that shows a dip or a peak when the photons are temporally indistinguishable (in), relative to the coincidence rate measured when temporally distinguishable (out). (**B**) The polarization distribution of input photons can be spatially tailored to engineer local variations in the quantum interference when overlapped on a 50:50 BS. The interference space-variant behavior is fully illustrated for three representative locations, depicted using distinct colors (blue, green, and pink). When tuning the temporal distinguishability, the polarization mixing in the quantum eraser results in a corresponding space-variant distribution of HOM, exhibiting peaks, dips, or flat curves. This takes place despite one photon being detected with a bucket detector.

The concept behind structuring quantum interference for tailoring photon coalescence is illustrated in Fig. 1. Although typical HOM scenarios imply identical photons, quantum interference can be also obtained when the two photons entering the BS are distinguishable, thanks to the quantum erasing process (*22*). When the which-path information is encoded in polarization, for instance, quantum interference can be enabled by placing a polarizer on each of the output paths, thus performing two projective measurements that erase the which-path information. Depending on the initial polarization state and each polarizer’s orientation, it is possible to fully tune photon coalescence from bunching to antibunching (Fig. 1A) (*2*). We could therefore exploit this feature to generate a structured quantum state with a tailored coalescence in the transverse plane if we design a spatially dependent quantum eraser. To this end, we consider a scenario where the polarization of each, otherwise indistinguishable photon, is given a nonuniform distribution in the transverse plane before impinging on separate ports of a 50:50 BS. A polarizer is moreover placed in each of the output paths of the BS. By tailoring the polarization profile of each photon and selecting the polarizer’s orientation, we can have full control over the spatial distribution of the photonic coalescence (Fig. 1B). In other words, by structuring quantum interference, photon bunching is tailored along the optical mode transverse profile so that in each position we can have a zero-or-two photon NOON state (HOM dips), a single photon (HOM peak), or some combination of the two. An interesting perspective is to consider the photon wave-particle properties associated with knowledge of the which-path information (*22*). By structuring the erasing process, we show that it is then possible to control this wave-particle behavior along the transverse profile of the optical mode.

### Experimental implementation

**Fig. 2.**
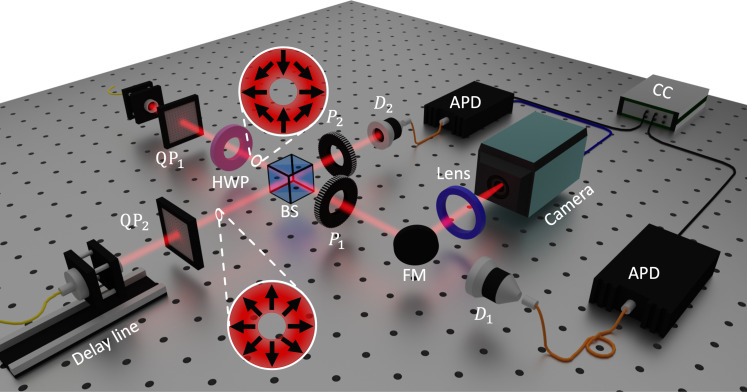
Experimental setup. After the spatial filtering with single mode fibers, two photons (λ = 810 nm), one in each path, are sent into two different input ports of a 50:50 BS as in a typical HOM setting. However, before reaching the BS, each of the photons is prepared in the desired VV mode (with spatial profiles shown as insets) by sending horizontally polarized light through *q* plates (QPs) with *q* = 0.5. Placement of an additional half-waveplate (HWP) after QP_1_ transforms one of the photons to the π mode. The temporal delay between the two photons is controlled by a translation stage mounted in the arm containing QP_2_. To realize bucket detection, the output photons are coupled (D_1_,D_2_) into multimode fibers and sent to two avalanche photodiodes (APD) connected to a coincidence counter (CC). For spatially resolved detection, one output is redirected via a flip mirror (FM) onto an intensifier coupled to a time-resolving pixel array (camera) and time-stamped with respect to the detection of the other photon (D_2_). Which-path projections are performed by rotating linear polarizers *P*_1_ and *P*_2_.

We experimentally demonstrate this concept by using the setup shown in Fig. 2, with full details provided in Materials and Methods. Initially, biphotons (λ = 810 nm) generated via spontaneous parametric down-conversion (SPDC), are separated in path and spatially filtered with single mode fibers. A set of polarization correcting waveplates (not shown in Fig. 2) facilitates uniform control of each photon’s polarization before being directed to the input ports of a 50:50 BS for interference, giving the two-photon state ${|\psi \rangle }_{\text{in}}=\left[{\hat{a}}_{A,m}^{†}\left(\tau \right){\hat{a}}_{B,n}^{†}\left(\tau^{\prime }\right)\right]|0\rangle$, where ${\hat{a}}^{†}$ is the creation operator for the respective modes, (*n*,*m*), with the time bins τ and τ′, in the discrete paths {*A*, *B*} of each input port. A motorized path delay is placed in one arm for tuning the temporal matching (τ′ → τ) of the photons. Initial matching of the input polarization replicates the standard HOM experiment when tuning the delay. Further insertion of geometric phase elements, known as *q* plates (QP) (*50*), before the BS allows the controlled fashioning of spatially varying polarization of the photons in each arm, dependent on the topological charge (*q*) of the element. This allows one to prepare a transverse mode (*n*) of the form

$$E_{n}\left(r,t\right)=e_{s}\left(\varphi \right)f\left(r,t-\tau \right)e^{-i\omega t}$$ (1)

for each photon, where *f* indicates the amplitude profile generally dependent on the radial (*r*) coordinate, {**e***_s_*(φ)} are the transverse unit vectors specifying the local mode polarization as a function of the azimuthal angle φ, ω is the mean (carrier) frequency of the mode, and time is *t*.

In particular, without loss of generality, we consider two modes that present the same polarization in some regions of the transverse profile, as depicted in Fig. 1B. As such, we choose the radial (*q* = 0.5) and π [*q* = 0.5 followed by a half-waveplate (HWP)] modes, which are two first-order orthogonal VV modes (*39*), to be the input states of the BS. These may respectively be described by the azimuthally dependent polarizations

$$\begin{array}{c} e_{\text{rad}}\left(\varphi \right)=e_{H}\text{cos}\varphi +e_{V}\text{sin}\varphi \\ e_{\pi }\left(\varphi \right)=e_{H}\text{cos}\varphi -e_{V}\text{sin}\varphi , \end{array}$$ (2)

where **e**_H_ and **e**_V_ are, respectively, the horizontal and vertical polarization unit vectors.

As in typical HOM experiments, the interference or coalescent behavior of our modes is observed through the twofold coincidences measured on the state

$${|\psi \rangle }_{\text{out}}=\frac{1}{2}\left[{\hat{a}}_{C,\text{rad}}^{†}\left(\tau \right){\hat{a}}_{D,\pi }^{†}\left(\tau^{\prime }\right)-{\hat{a}}_{C,\pi }^{†}\left(\tau^{\prime }\right){\hat{a}}_{D,\text{rad}}^{†}\left(\tau \right)\right]|0\rangle$$ (3)

corresponding to the projection of the full BS-output state onto the subspace in which the two photons are separated in ports C and D, and may be calculated by the fourth-order correlation function (*51*), as detailed in the Supplementary Materials. With this, we find the spatially varying coincidence probabilities for arbitrary polarization projections {α,β} on the photons detected in each port (respectively corresponding to coordinates φ_1_ and φ_2_) when temporally tuned (τ = τ′)

$$\begin{array}{cc} C_{\left(\text{In}\right)}^{\alpha ,\beta }\left(\varphi_{1};\varphi_{2}\right)= & \frac{1}{4}|\left[u_{\alpha }\cdot e_{\text{rad}}\left(\varphi_{1}\right)\right]\left[u_{\beta }\cdot e_{\pi }\left(\varphi_{2}\right)\right]\\ & -{\left[u_{\alpha }\cdot e_{\pi }\left(\varphi_{1}\right)\right]\left[u_{\beta }\cdot e_{\text{rad}}\left(\varphi_{2}\right)\right]|}^{2} \end{array}$$ (4)

and temporally distinguishable (τ ≠ τ′)

$$\begin{array}{cc} C_{\left(\text{Out}\right)}^{\alpha ,\beta }\left(\varphi_{1};\varphi_{2}\right)= & \frac{1}{4}\left\{{|\left[u_{\alpha }\cdot e_{\text{rad}}\left(\varphi_{1}\right)\right]\left[u_{\beta }\cdot e_{\pi }\left(\varphi_{2}\right)\right]|}^{2}\right.\\ & \left.+{|\left[u_{\alpha }\cdot e_{\pi }\left(\varphi_{1}\right)\right]\left[u_{\beta }\cdot e_{\text{rad}}\left(\varphi_{2}\right)\right]|}^{2}\right\} \end{array}$$ (5)

Here **u**_α_ and **u**_β_ are the unit vectors directed along the detection polarizer axes. For simplicity, we ignore the radial distribution as the polarization variation depends only on φ (see the Supplementary Materials for a more general treatment). Experimentally, we realize these polarization state projections by respectively placing polarizers *P*_1_ and *P*_2_ in each output port of the BS.

As depicted in Fig. 2, we adopted two strategies to observe the generated structure by means of these coincidences, both in and out of the temporal tuning. In one case, the photons from each output port were collected by coupling into multimode fibers connected to bucket detectors placed in each arm (flip mirror down), rendering no resolution of the spatial distribution. This corresponds to measuring the correlations given in Eqs. 4 and 5 after integrating over both azimuthal angles φ_1_ and φ_2_. In the second case (flip mirror up), we performed spatially resolved measurements of one photon by replacing one bucket detector with a camera, which was then conditioned on the spatially unresolved detection of the other photon by bucket detection in the other arm. This corresponds to measuring the correlations given in Eqs. 4 and 5 after integrating over one angle only, e.g., φ_2_.

It is now possible to identify polarization projection measurements that erase the which-path information and enable quantum interference between these structured modes, even for bucket detection. Intuitively, one may consider the VV spatial distributions depicted in Fig. 2, which shows that they share the same polarization state along the horizontal (H) and vertical (V) axes, while being completely orthogonal along the diagonal (D) and antidiagonal (A) directions. It thus follows that a projection along H and V should destroy the which-path information for the latter pairing and recover HOM interference. To observe the related coalescence for such polarizations (α and β), we use a visibility (*52*), defined as $V_{\alpha ,\beta }=\left(C_{\text{out}}^{\alpha ,\beta }-C_{\text{in}}^{\alpha ,\beta }\right)/C_{\text{out}}^{\alpha ,\beta }\in \left[-1,1\right]$ between the coincidences detected out (*C*_out_) and in (*C*_in_) the temporal indistinguishably criterion (as depicted in Fig. 1B). This allows us to easily recognize bunching as V becomes positive from antibunching where V becomes negative with extreme cases of perfect bunching (antibunching) being detected when V = 1 (−1).

**Fig. 3.**
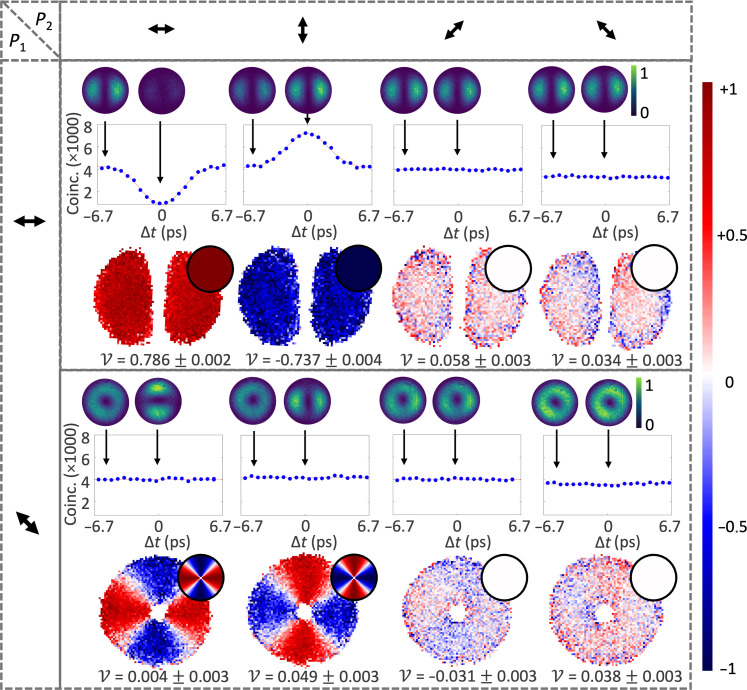
Spatially tailored photon distributions from quantum interference. Table of experimental outcomes for different choices of polarization projections *P*_1_ (black arrows, row-wise) and *P*_2_ (black arrows, column-wise). HOM coincidence counts measured without spatial resolution and varying the temporal delay are shown as blue points in the upper graphs of each panel (solid curves correspond to best fits). Error bars from Poissonian detection statistics are smaller than data points. Spatially resolved coincidence images (spanning ∼70 × 70 pixels) observed with the camera are given as insets above the HOM curves with arrows denoting the temporally tuned (Δ*t* = 0, In) measurement to the right and temporally distinguishable (Δ*t* ∼ −6 ps, Out) measurement to the left. False colors indicate the intensity of coincidences, individually normalized for each measurement set (in and out the dip). The related spatially resolved visibilities are given as the transverse distributions at the bottom of each panel, using the false-color scale shown on the right-side bar. Integrated visibilities for each distribution are indicated below and analytically calculated visibility distributions given as top-right circular insets. All results have been corrected for background noise (see Materials and Methods for details and Supplementary Materials for raw data).

The line plots given in each panel of Fig. 3 show the experimental outcomes of bucket-only detection as the delay between the two input photons was varied by moving the delay line. Here, projections (*P*_1_, *P*_2_) = (H,H) elicit a dip (V = 0.81 ± 0.02) at Δ*t* = 0, coinciding with an erasure of the which-path information for the two photons and thereby restoring quantum interference. For (*P*_1_, *P*_2_) = (H,V), we find a peak instead of a dip (V = −0.74 ± 0.09), corresponding to photon antibunching (*2*, *11*). Alternatively, all cases involving a projection along D and/or A, result in a flat curve (no HOM dip or peak).

On the other hand, for spatially resolvable measurements (using the camera), we expect the following spatially structured visibilities, corresponding to a set of polarization projections of *P*_1_ ∈ {H, A} in the camera arm and *P*_2_ ∈ {H, V, D, A} in the bucket detector arm as depicted by the arrows in Fig. 3

$$\begin{array}{c} V_{\text{HH}}=1\\ V_{\text{HV}}=-1\\ V_{\text{AH}}=\text{cos}\left(2\varphi_{1}\right)\\ V_{\text{AV}}=-\text{cos}\left(2\varphi_{1}\right)\\ V_{\text{HA}}=V_{\text{HD}}=V_{\text{AA}}=V_{\text{AD}}=0 \end{array}$$ (6)

obtained by integrating Eqs. 4 and 5 over φ_2_ due to bucket detection in arm 2 (see the Supplementary Materials for more details). In this case, images captured in coincidence with the bucket detector (see Materials and Methods) were measured for “out” (upper left inset of the curves) and “in” (upper right inset of the curve) the temporally matched condition. Each measured pair of coincidence distributions then allowed us to reconstruct the visibility map (lower row) for the projection settings.

The resulting outcomes relate well to what was observed in the case of no spatial resolution (bucket detection) as the coincidences measured by bucket detection are proportional to the integrated pixel distribution over the mode profile. We additionally compare these to the calculated profiles from Eq. 6 that are shown as upper-right insets of the visibility maps. Accordingly, (*P*_1_, *P*_2_) = (H,H) shows uniform coalescence across the spatial distribution, while (H,V) exhibits a uniform anticoalescence, within the expectations of the calculated map. The cases of (H,A), (H,D), (A,A), and (A,D) similarly yield a uniform structure in line with expectations of zero visibility across the measured profile. Faint residual structures in these cases are ascribed to experimental imperfections, such as optical misalignments or mode imperfections, nonetheless remaining close to 0.

**Fig. 4.**
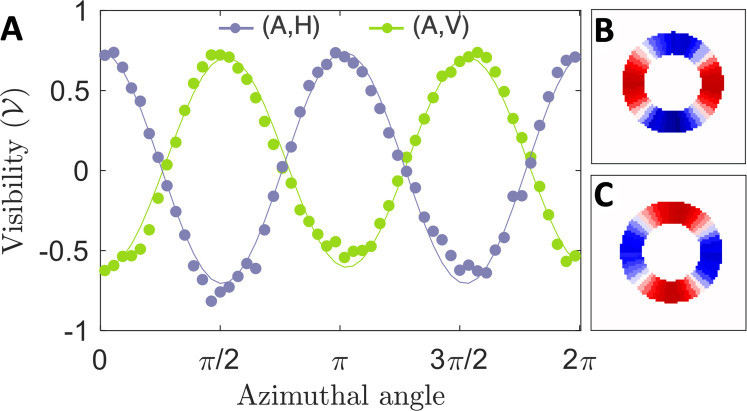
Spatially varying coalescence. (**A**) Visibility cross sections showing the mean visibility across 54 radial wedges as they vary azimuthally along the spatial visibility maps shown in Fig. 3. Error bars from Poissonian detection statistics are too small to observe. Plots of the visibility distribution averaged over wedges for (**B**) (A,V) and (**C**) (A,H) eraser projections used to calculate the circular visibility profiles. Best-fit sinusoidal curves are included as solid lines to guide the eye.

Crucially, projections (A,H) and (A,V) reveal spatially varying features with two complementary lobes, showing coalescence and anticoalescence across the transverse plane as predicted by Eq. 6. These structures are not detectable without spatial resolution, as shown by the flat curves in the corresponding graphs. We further illustrate this variation in Fig. 4A by plotting visibilities calculated along a circular cross-section divided into wedges within the region of strong signal (see Materials and Methods for more detail). A clear variation occurs azimuthally from bunched where V = 0.738 ± 0.003 (V = 0.738 ± 0.003) to antibunched with V = −0.816 ± 0.003 (V = −0.625 ± 0.003) for the (A,H) [(A,V)] spatial distribution. Figure 2 (B and C) shows the averaged experimental wedges, respectively processed to give the (A,H) and (A,V) curves. As a result, we herald a mode of structured bosonic coalescence with some spatial regions containing either zero or two photons (red) as in the case of NOON states. At the same time, other regions of the mode, corresponding to anticoalescence, will always be populated by one photon (blue).

## DISCUSSION

Our results highlight interesting features of structured quantum light, based on the projections made in either arm. For instance, a diagonal projection only revealed structurally varying coalescence when in the arm of the camera and one only observed nonzero visibilities for H and V polarizations projections in the arm of the bucket detector. To understand this, one must consider that polarization is paired to spatial structure in VV modes. In our case, projection on H or V causes both photons to have identical spatial modes (lobes). This completely erases the which-path information, giving rise to interference (visibility). Measurement settings A and D, however, leave this spatial degree of freedom in orthogonal modes, preserving which-path information in spatially integrated measurements with the multimode fiber. As a result, bucket detection for A and D projections gives null visibilites for all cases. Conversely, while these modes are “globally” orthogonal, they have local regions of overlap that vary with spatial position, yielding a local indistinguishability not seen without spatial resolution of the detector. Bucket detection with H and V then allows these locally indistinguishable structures for A and D to be observed. Furthermore, a distinct reversal of the coalescent and anticoalescent distributions can be seen for complementary polarization projections. One can thus locally switch between bosonic and fermionic behavior, or equivalently, between two/zero (NOON) and single photon occupation. It follows that tailoring the quantum state can be conveniently achieved by changing the polarization distribution of the input beams as well as the polarization projections.

In conclusion, we have generated structured quantum states of light that have no classical counterpart, with a spatially tailored bosonic coalescence. Structured light typically refers to classical electromagnetic modes, where one or more properties of light, such as intensity distribution, polarization distribution, or temporal shape, are tailored and controlled by the user. These degrees of freedom are available in both the classical and quantum regimes; for instance, we can have a single photon in a VV mode or a classical vector beam. Instead, here, we develop a technique to tailor quantum correlations between photons. In our experiment we considered two photons and obtained a biphoton structured state with no classical counterpart in the sense discussed above. We achieved this by exploiting a simple and robust scheme based on spatially tailored distinguishability between photons in a quantum eraser setting. As a result we were able to control the wave-particle behavior of photons along the transverse profile of the optical mode. This additionally invites insight into the structured state where, in particular, the use of orthogonal modes for each interfered photon showed a global versus local HOM effect (*6*) such that detection without spatial resolution yielded no sign of interference, but spatially resolved measurements reveal its presence. Although these results were demonstrated for VV modes, our technique can be generalized to engineer arbitrary desired interference structures for varying purposes and using different vectorial modes, including hybrid modes (*53*, *54*). In addition, we point out that programmable approaches for tailoring the input polarization distributions (*55*) would allow dynamic on-demand variations of these states. As distinguishability is the property one needs to tailor, one may extend our result to other degrees of freedom, such as temporally structured light (*56*) as well as multiparticle scenarios (*57*). By providing a robust and flexible way to engineer correlations, our scheme could find applications in photonic quantum computation tasks based on multiphoton/multi-interference protocols (*58*) or in quantum communication scenarios where users can share images encoded in quantum correlation of a multiphoton state (*59*). Last, we believe the structured quantum eraser concept could be beneficial in fields where photon coalescence and structured light are crucial resources such as quantum microscopy, sensing, and metrology.

## MATERIALS AND METHODS

### Detailed experimental setup

The two (λ = 810 nm) signal-idler photons used in the experiment (Fig. 2) for interference on the BS were generated in the state ∣ψ〉 = ∣H〉∣V〉 via type II degenerate collinear SPDC from a 30-mm-long ppKTP crystal, which provided high efficiencies and controlled phase-matching. The crystal was pumped with a λ = 405 nm continuous-wave laser and temperature phase matched at ∼38°C. A spectral width of ∼0.2 nm was estimated for the photons from the HOM dip, matching our numerical calculations, and no bandpass filtering was used. The photons were then path separated with a polarizing BS and each spatially filtered by coupling into single-mode fibers (SMF) which directed the photons through an HWP and quater-waveplate (for preparation into the horizontal polarization state) as well as electronically tuned QPs with the topological charge *q* = 0.5 for polarization-dependent spatial structuring, before impinging onto a 50:50 BS for interference. One SMF was placed on a motorized translation stage to introduce a path delay for temporally tuning the distinguishability. The output of each BS port was then directed toward detectors. We switched between space-integrated and space-resolved measurements by using a flip mirror. The first case (flip mirror down) coupled the structured photons in each arm into multimode fibers for detection by single-photon counting modules [avalanche photodiode (APD)] with ∼60% efficiency connected to a coincidence counter. In the second case, the photons in one arm were diverted by the flip mirror onto an intensified TimePix3 camera with ∼19% efficiency and time stamped alongside the remaining APD signal using built-in electronics. Polarization projections on the structured states were made by inserting linear polarizers into each output arm before the relevant detectors.

### Data acquisition and analysis

Measurements using both APDs were obtained for an integration time of 1 s and a coincidence window of 2 ns. Each spatial measurement with the TimePix3 captured coincidences within a 20-ns window and integrated over 13.3 min using 10 frames of 80-s exposure each. The data acquired reported the time stamp at which the counts for each pixel were detected alongside the counts received by the APD. A clustering algorithm was used to extrapolate the spread in signal across the pixels from the amplification provided by a PhotonisIR intensifier to identify photons [see (*60*–*62*) for more information]. For both the HOM and spatially resolved measurements, noise (dark counts and accidentals) was measured by observing the counts with an additional large delay (20 and 1000 ns, respectively) between the two signals so as to be outside of the coincidence window. This noise floor was then removed from the coincidence signal to produce the plots presented in Fig. 3. Although background subtraction could introduce loopholes that need to be taken into account for some applications and quantum tests (*63*, *64*), such data processing is commonly performed in camera based quantum experiments to improve signal-to-noise ratio (*65*–*72*). Furthermore, our spatially integrated visibilities for background-subtracted images are in close agreement with the corresponding fiber-only measurements for which background was not as relevant, therefore further supporting the validity of our background subtraction protocol. An additional region of interest condition was applied to the spatial images in the visibility map calculation where areas without signal (3σ inside the noise floor) were excluded.

The average counts detected in output arms containing polarizer *P*_1_ (*P*_2_) were 216 ± 16 (211 ± 17) *k*-counts per second with the APD and 71 ± 2 *k*-counts per second with the TimePix3 camera for all projection settings. Across the spatial distributions, there was an average of 212.4 coincidences per pixel for the 13.3 min measurement with an average background noise of 92.0 counts per pixel, of which, 37.5 were dark counts. An average contrast (*C* = signal/noise) outside the dip of *C* = 2.30 was obtained for the images and *C* = 15.4 for the APD-only measurements. The azimuthally changing visibility distributions (AH and AV configurations) were investigated by splitting the measured images into 54 circular sectors (wedges), where the center was calculated by geometric averaging the “out of the dip” coincidences distribution, and calculating the visibility for each sector.

### VV modes

In the experiment, VV modes were generated by exploiting spin-to-orbital angular momentum conversion in a birefringent liquid crystal slab with uniform retardation and an azimuthally varying optical axis, known as a QP. This device is characterized by a topological charge, *q*, related to the number of rotations of the local optical axis and, accordingly, the polarization-dependent geometric phase imparted to incident light. In Jones matrix formalism, the QP operation in the linear basis, where ∣H〉 = [1; 0]*^T^* and ∣V〉 = [0; 1]*^T^* are horizontal and vertical polarization states, is described as

$$\text{QP}=\left[\begin{array}{cc} \text{cos}\left(2q\varphi \right) & \text{sin}\left(2q\varphi \right)\\ \text{sin}\left(2q\varphi \right) & -\text{cos}\left(2q\varphi \right) \end{array}\right]$$ (7)

From this, it is straightforward to see the spatially varying polarization distribution induced and dependence on polarization. Taking a horizontally polarized state as in the experiment, one obtains the following output

$$\text{QPH}=\left[\begin{array}{cc} \text{cos}\left(2\mathrm{q\varphi}\right) & \text{sin}\left(2\mathrm{q\varphi}\right)\\ \text{sin}\left(2\mathrm{q\varphi}\right) & -\text{cos}\left(2\mathrm{q\varphi}\right) \end{array}\right]\left[\begin{array}{c} 1\\ 0 \end{array}\right]=\left[\begin{array}{l} \text{cos}\left(2\mathrm{q\varphi}\right)\\ \text{sin}\left(2\mathrm{q\varphi}\right) \end{array}\right]$$ (8)

and with *q* = 0.5 as in the experiment, the state described in Eq. 2 is obtained. By adding an HWP = [1,0; 0, −1]*^T^* where the optical axis aligned horizontally with the QP, the following transformation is achieved

$$\begin{array}{cc} \left[\text{HWP}\right]\left[\text{QP}\right] & =\left[\begin{array}{cc} 1 & 0\\ 0 & -1 \end{array}\right]\left[\begin{array}{cc} \text{cos}\left(2\mathrm{q\varphi}\right) & \text{sin}\left(2\mathrm{q\varphi}\right)\\ \text{sin}\left(2\mathrm{q\varphi}\right) & -\text{cos}\left(2\mathrm{q\varphi}\right) \end{array}\right]\\ & =\left[\begin{array}{cc} \text{cos}\left(2\mathrm{q\varphi}\right) & \text{sin}\left(2\mathrm{q\varphi}\right)\\ -\text{sin}\left(2\mathrm{q\varphi}\right) & \text{cos}\left(2\mathrm{q\varphi}\right) \end{array}\right] \end{array}$$ (9)

so that, acting on the state *H*, the desired state ∣π〉 = cos (φ)∣H〉 − sin (φ)∣V〉 is produced for *q* = 0.5.

## Supplementary Materials

### This PDF file includes:

Supplementary Text Figs. S1 and S2 References
