# Supplementary material for: Engineering quantum states from a spatially structured quantum eraser
Source: Sci Adv. 2024 Jul 24;10(30):eadm9278. doi: 10.1126/sciadv.adm9278 (PMC11268414; doi:10.1126/sciadv.adm9278)
Supplement: Supplementary file 1 — Supplementary Text Figs. S1 and S2 References [file sciadv.adm9278_sm.pdf]

Science Advances
AAAS

Supplementary Materials for

Engineering quantum states from a spatially structured quantum eraser

Carlo Schiano *et al.*

Corresponding author: Vincenzo D'Ambrosio, vincenzo.dambrosio@unina.it

Sci. Adv. **10**, eadm9278 (2024)
DOI: 10.1126/sciadv.adm9278

This PDF file includes:

Supplementary Text
Figs. S1 and S2
References

Supplementary Information: Engineering quantum states from a spatially structured quantum eraser

Carlo Schiano,[1] Bereneice Sephton,[1] Roberto Aiello,[1] Francesco Graffitti,[2] Nijil Lal,[1] Andrea Chiuri,[3] Simone Santoro,[3] Luigi Santamaria Amato,[4] Lorenzo Marrucci,[1,5] Corrado de Lisio,[1] and Vincenzo D'Ambrosio[1]

[1]Dipartimento di Fisica, Università di Napoli Federico II, Complesso Universitario di Monte S. Angelo, Via Cintia, 80126 Napoli, Italy

[2]Institute of Photonics and Quantum Sciences, School of Engineering and Physical Sciences, Heriot-Watt University, Edinburgh EH14 4AS, UK

[3]Enea - Centro Ricerche Frascati, via E. Fermi 45, 00044 Frascati, Italy

[4]Italian Space Agency (ASI), Centro di Geodesia Spaziale 'Giuseppe Colombo', Località Terlecchia, 75100 Matera, Italy

[5] CNR-ISASI, Institute of Applied Science and Intelligent Systems, Via Campi Flegrei 34, 80078 Pozzuoli (NA), Italy

1. STRUCTURAL CORRELATION FORMULATION

We send two structured photon beams into a beamsplitter (BS) and measure two-fold coincidence between a bucket detector and a camera, after polarisation projection. We record different spatial structures on the camera, depending on the polarisation projection performed and on the choice of the input structured beams. Here we describe in detail how it works with theoretical calculations.

According to the quantum theory of electrodynamics, the transverse electric field operator can be written as [73]:

$$\hat{\mathbf{E}}_{\perp}(\mathbf{r},t) = \hat{\mathbf{E}}_{\perp}^{+}(\mathbf{r},t) + \hat{\mathbf{E}}_{\perp}^{-}(\mathbf{r},t) \tag{S1}$$

where $\hat{\mathbf{E}}_{\perp}^{+}(\mathbf{r},t) = \sum_n \mathbf{E}_n(\mathbf{r},t)\hat{a}_n$ and $\hat{\mathbf{E}}_{\perp}^{-}(\mathbf{r},t) = \sum_n \mathbf{E}_n^{*}(\mathbf{r},t)\hat{a}_n^{\dagger}$ are the positive and negative frequencies field components, respectively. The index n spans a complete set of normal modes $\{\mathbf{E}_n(\mathbf{r},t)\}$, and $\hat{a}_n$ and $\hat{a}_n^{\dagger}$ are the photon annihilation and creation operators, respectively, with the usual commutation rules $\left[\hat{a}_n, \hat{a}_{n'}^{\dagger}\right] = \delta_{n,n'}$. We are here assuming for convenience that these modes fill a finite space volume Ω and satisfy suitable boundary conditions at the volume border so as to discretize the volume set.

For any given quantum state $|\psi\rangle$ (we assume pure quantum states here, but the treatment can be easily generalized to the general case of quantum mixtures), the number of double detections per unit of time between polarised photons hitting two separate broad-band pointlike detector is proportional to the fourth-order correlation function [51]

$$w_{II}^{\alpha,\beta}(\mathbf{r}_1,t_1;\mathbf{r}_2,t_2) = \langle\psi| \left[\mathbf{u}_\alpha \cdot \hat{\mathbf{E}}_{\perp}^{-}(\mathbf{r}_1,t_1)\right] \left[\mathbf{u}_\beta \cdot \hat{\mathbf{E}}_{\perp}^{-}(\mathbf{r}_2,t_2)\right] \left[\mathbf{u}_\beta \cdot \hat{\mathbf{E}}_{\perp}^{+}(\mathbf{r}_2,t_2)\right] \left[\mathbf{u}_\alpha \cdot \hat{\mathbf{E}}_{\perp}^{+}(\mathbf{r}_1,t_1)\right] |\psi\rangle \tag{S2}$$

where $\mathbf{u}_\alpha$ and $\mathbf{u}_\beta$ are the unit-vectors corresponding to the polarizations being detected at r_1 and r_2, respectively.

For our purposes, it is convenient to choose a set of modes that are eigenstate of vector beams, described by the OAM eigenvalue m ($L_z = m\hbar$) and by a non-negative integer p specifying different orthogonal radial modes for each $|m|$. In our case, we consider quasi-monochromatic pulsed modes with mean (carrier) frequency ω, belonging to discrete paths $P \in \{A, B\}$ and having a specified time bin τ (so that delay can be used to distinguish the photons). In the paraxial approximation, assuming that z is the main propagation axis, these modes can be written as follows:

$$\mathbf{E}_n(\mathbf{r},t) = \mathbf{e}_s f_{|m|,p}(r_P, z_P, t-\tau)e^{im\varphi_P}e^{-i\omega t} \tag{S3}$$

where (r_P, z_P, φ_P) are cylindrical coordinates for the given path P, $f_{|m|,p}$ specifies the radial profile of the mode amplitude as a function of z, and $\{\mathbf{e}_s\}$ are two unit-vectors orthogonal to z with polarisation index s. Thus, the mode index can be written as $n = (P, m, p, \omega, \tau, s)$. We assume that different values of the various indices correspond to orthogonal modes. Rather than describing the vector beams as superpositions of modes like in Eq. (S3), it is also

possible (and in our case simpler) to introduce in Eq. (S3) an azimuthally-position-dependent polarisation, $\mathbf{e}_s(\varphi)$ (in this case the m integer is not the OAM eigenvalue anymore). In our experiment, the vector beams impinging on the faces of the BS are the radial and π beam (with total angular momentum 2 and OAM 1), that are respectively described by the following azimuthally-position-dependent polarisations

$$\begin{aligned} \mathbf{e}_{rad}(\varphi) &= \mathbf{e}_H \cos\varphi + \mathbf{e}_V \sin\varphi \\ \mathbf{e}_{\pi}(\varphi) &= \mathbf{e}_H \cos\varphi - \mathbf{e}_V \sin\varphi \end{aligned} \tag{S4}$$

where e_i, $i \in \{H, V\}$ are the horizontal (H) and vertical (V) polarisation unit-vector. Let us now consider certain more specific states, obtained by having two photons in well-defined vector beams (vv1 and vv2) impinging on the two input faces (A and B) of a beamsplitter (BS) and exiting from the two output faces (C and D). We assume that all properties of the photon beams, except their time bins, are identical (we omit them for brevity), so that detected photons become indistinguishable only if $\tau = \tau'$. By making these assumptions, the quantum state $|\psi\rangle$ in Eq. (S2) can be obtained by acting with a 50:50 beamsplitter on the two-photon state $|\psi\rangle_{in} = a^{\dagger}_{A,vv1}(\tau) a^{\dagger}_{B,vv2}(\tau') |0\rangle$ and thus it is given by

$$|\Psi\rangle = \frac{1}{2}\left(\hat{a}^{\dagger}_{C,vv1}(\tau) + i\hat{a}^{\dagger}_{D,vv1}(\tau)\right)\left(i\hat{a}^{\dagger}_{C,vv2}(\tau') + \hat{a}^{\dagger}_{D,vv2}(\tau')\right)|0\rangle \tag{S5}$$

where $a^{\dagger}$ is the creation operator for the $vv1$ and $vv2$ modes, with the time-bins, τ and τ', in the discrete paths $\{C, D\}$ of each BS port. Because we are only interested in the coincidences obtained when placing one detector in path C and the other in path D, we may now project (or "postselect") this state within the subspace in which the two photons are separated in ports C and D (the other cases do not give coincidences). We thus obtain the following expression for the unnormalized (projected) quantum state

$$|\Psi\rangle = \frac{1}{2}\left[\hat{a}^{\dagger}_{C,vv1}(\tau)\hat{a}^{\dagger}_{D,vv2}(\tau') - \hat{a}^{\dagger}_{C,vv2}(\tau')\hat{a}^{\dagger}_{D,vv1}(\tau)\right] \tag{S6}$$

that corresponds to the two-photon input state of Eq. (3) in the main text (if we put $vv1 = rad$ and $vv2 = \pi$). Inserting Eq. (S6) into Eq. (S2) and exploiting the creation-annihilation operator commutation rules, we immediately obtain the expected fourth-order correlation function

$$\begin{aligned} w^{\alpha,\beta}_{II}(\mathbf{r}_1, t_1; \mathbf{r}_2, t_2) = \frac{1}{4}\,&|[\mathbf{u}_\alpha \cdot \mathbf{E}_{C,vv1,\tau}(\mathbf{r}_1, t_1)]\,[\mathbf{u}_\beta \cdot \mathbf{E}_{D,vv2,\tau'}(\mathbf{r}_2, t_2)] - \\ &[\mathbf{u}_\alpha \cdot \mathbf{E}_{C,vv2,\tau'}(\mathbf{r}_1, t_1)]\,[\mathbf{u}_\beta \cdot \mathbf{E}_{D,vv1,\tau}(\mathbf{r}_2, t_2)]|^2 \end{aligned} \tag{S7}$$

If we now insert the modes expression Eq. (S3) into Eq. (S7), we obtain

$$\begin{aligned} w^{\alpha,\beta}_{II}(\mathbf{r}_1, t_1; \mathbf{r}_2, t_2) = \frac{1}{4}\,&|[\mathbf{u}_\alpha \cdot \mathbf{e}_{vv1}(\varphi_1) f(r_1, z_1, t_1 - \tau)]\,[\mathbf{u}_\beta \cdot \mathbf{e}_{vv2}(\varphi_2) f(r_2, z_2, t_2 - \tau')] - \\ &[\mathbf{u}_\alpha \cdot \mathbf{e}_{vv2}(\varphi_1) f(r_1, z_1, t_1 - \tau')]\,[\mathbf{u}_\beta \cdot \mathbf{e}_{vv1}(\varphi_2) f(r_2, z_2, t_2 - \tau)]|^2 \quad . \end{aligned} \tag{S8}$$

In order to get the coincidence probability between two photons whose polarisation are directed along $\mathbf{u}_\alpha$ and $\mathbf{u}_\beta$, this rate must be integrated both in t_1 and t_2 over the same detection time window

$$C_{\alpha,\beta}(\mathbf{r}_1; \mathbf{r}_2) = \int w^{\alpha,\beta}_{II}(\mathbf{r}_1, t_1; \mathbf{r}_2, t_2)\, dt_1 dt_2 \tag{S9}$$

We must distinguish here the case of temporally tuned photons ($\tau = \tau'$) from the case of temporally distinguishable photons ($\tau \neq \tau'$). In the former case, we are reproducing the "in the dip/peak" condition of a typical HOM experiment, and we obtain

$$C^{\alpha,\beta}_{(In)}(\mathbf{r}_1; \mathbf{r}_2) = \frac{F(r_1, z_1)F(r_2, z_2)}{4}|[\mathbf{u}_\alpha \cdot \mathbf{e}_{vv1}(\varphi_1)]\,[\mathbf{u}_\beta \cdot \mathbf{e}_{vv2}(\varphi_2)] - [\mathbf{u}_\alpha \cdot \mathbf{e}_{vv2}(\varphi_1)]\,[\mathbf{u}_\beta \cdot \mathbf{e}_{vv1}(\varphi_2)]|^2 \tag{S10}$$

while, in the latter case, assuming that both time bins fall in the integration window but do not overlap, we are reproducing the "out the dip/peak" condition of a typical HOM experiment, and we obtain

$$C^{\alpha,\beta}_{(Out)}(\mathbf{r}_1; \mathbf{r}_2) = \frac{F(r_1, z_1)F(r_2, z_2)}{4}\left\{|[\mathbf{u}_\alpha \cdot \mathbf{e}_{vv1}(\varphi_1)]\,[\mathbf{u}_\beta \cdot \mathbf{e}_{vv2}(\varphi_2)]|^2 + |[\mathbf{u}_\alpha \cdot \mathbf{e}_{vv2}(\varphi_1)]\,[\mathbf{u}_\beta \cdot \mathbf{e}_{vv1}(\varphi_2)]|^2\right\} \tag{S11}$$

where we introduced the "fluence" function

$$F(r,z) = \int\limits_{\substack{\text{integration}\\ \text{window}}} f^2(r,z,t-\tau)dt \tag{S12}$$

By setting $vv1 = rad$ and $vv2 = \pi$ and ignoring the radial distribution (as the polarisation variation depends only on φ), Eqs. (S10) and (S11) reduce to Eqs. (4) and (5) in main text, respectively. In our experiment, the coincidence probability functions are measured by considering $vv1 = rad$ and $vv2 = \pi$ vector beams impinging the BS and by setting the polarisation $P_1 \in \{H, A\}$ for the polariser located before the camera and $P_2 \in \{H, V, A, D\}$ for the polariser located before the bucket detector, where $\hat{e}_D = (\hat{e}_H + \hat{e}_V)/\sqrt{2}$ and $\hat{e}_A = (\hat{e}_H - \hat{e}_V)/\sqrt{2}$ are the unit-vectors associated to D and A, respectively. We are interested in theoretically evaluating the visibility map given by the formula

$$\mathcal{V}_{\alpha,\beta}(\mathbf{r}_1;\mathbf{r}_2) = \frac{C^{\alpha,\beta}_{(Out)}(\mathbf{r}_1;\mathbf{r}_2) - C^{\alpha,\beta}_{(In)}(\mathbf{r}_1;\mathbf{r}_2)}{C^{\alpha,\beta}_{(Out)}(\mathbf{r}_1;\mathbf{r}_2)} \tag{S13}$$

The visibility map is calculated for the 8 different polarisation combinations listed above and the results are presented below. We set $A_{1,2} = F(r_1,z_1)F(r_2,z_2)$ in order to lighten up the notation in the following equations. In case of H-H and H-V polarisation settings, the visibility map does not depend on the azimuthal angles φ_1 and φ_2.

$$\left.\begin{aligned} C^{H,H}_{(Out)}(\mathbf{r}_1;\mathbf{r}_2) &= \frac{A_{1,2}}{2}\cos^2\varphi_1\cos^2\varphi_2 \\ C^{H,H}_{(In)}(\mathbf{r}_1;\mathbf{r}_2) &= 0 \end{aligned}\right\} \quad \mathcal{V}_{HH}(\varphi_1;\varphi_2) = 1 \tag{S14}$$

$$\left.\begin{aligned} C^{H,V}_{(Out)}(\mathbf{r}_1;\mathbf{r}_2) &= \frac{A_{1,2}}{2}\cos^2\varphi_1\cos^2\varphi_2{}^2 \\ C^{H,V}_{(In)}(\mathbf{r}_1;\mathbf{r}_2) &= A_{1,2}\cos^2\varphi_1\cos^2\varphi_2 \end{aligned}\right\} \quad \mathcal{V}_{HV}(\varphi_1;\varphi_2) = -1 \tag{S15}$$

In case of H-A, H-D, A-H, A-V polarisation settings, the visibility map depends only on the φ_2 angle for the H-A and H-D configurations, while it depends only on the φ_1 angle for the A-H and A-V configurations.

$$\left.\begin{aligned} C^{H,A}_{(Out)}(\mathbf{r}_1;\mathbf{r}_2) = C^{H,D}_{(Out)}(\mathbf{r}_1;\mathbf{r}_2) &= \frac{A_{1,2}}{4}\cos^2\varphi_1 \\ C^{H,A}_{(In)}(\mathbf{r}_1;\mathbf{r}_2) = C^{H,D}_{(In)}(\mathbf{r}_1;\mathbf{r}_2) &= \frac{A_{1,2}}{2}\cos^2\varphi_1\sin^2\varphi_2 \end{aligned}\right\} \quad \mathcal{V}_{HA}(\varphi_1;\varphi_2) = \mathcal{V}_{HD}(\varphi_1;\varphi_2) = \cos(2\varphi_2) \tag{S16}$$

$$\left.\begin{aligned} C^{A,H}_{(Out)}(\mathbf{r}_1;\mathbf{r}_2) &= \frac{A_{1,2}}{4}\cos^2\varphi_2 \\ C^{A,H}_{(In)}(\mathbf{r}_1;\mathbf{r}_2) &= \frac{A_{1,2}}{2}\sin^2\varphi_1\cos^2\varphi_2{}^2 \end{aligned}\right\} \quad \mathcal{V}_{AH}(\varphi_1;\varphi_2) = \cos(2\varphi_1) \tag{S17}$$

$$\left.\begin{aligned} C^{A,V}_{(Out)}(\mathbf{r}_1;\mathbf{r}_2) &= \frac{A_{1,2}}{4}\sin^2\varphi_2 \\ C^{A,V}_{(In)}(\mathbf{r}_1;\mathbf{r}_2) &= \frac{A_{1,2}}{2}\cos^2\varphi_1\sin^2\varphi_2 \end{aligned}\right\} \quad \mathcal{V}_{AV}(\varphi_1;\varphi_2) = -\cos(2\varphi_1) \tag{S18}$$

In case of A-A, A-D polarisation configurations, the visibility map depends on the φ_1 and the φ_2 angles, both.

$$\left.\begin{aligned} C^{A,A}_{(Out)}(\mathbf{r}_1;\mathbf{r}_2) &= \frac{A_{1,2}}{8}\left[\cos^2(\varphi_1-\varphi_2) + \sin^2(\varphi_1+\varphi_2)\right] \\ C^{A,A}_{(In)}(\mathbf{r}_1;\mathbf{r}_2) &= \frac{A_{1,2}}{4}\sin^2(\varphi_1+\varphi_2) \end{aligned}\right\} \quad \mathcal{V}_{AA}(\varphi_1;\varphi_2) = \frac{\cos^2(\varphi_1-\varphi_2) - \sin^2(\varphi_1+\varphi_2)}{\cos^2(\varphi_1-\varphi_2) + \sin^2(\varphi_1+\varphi_2)} \tag{S19}$$

$$\left.\begin{aligned} C^{A,D}_{(Out)}(\mathbf{r}_1;\mathbf{r}_2) &= \frac{A_{1,2}}{8}\left[\cos^2(\varphi_1+\varphi_2) + \sin^2(\varphi_1-\varphi_2)\right] \\ C^{A,D}_{(In)}(\mathbf{r}_1;\mathbf{r}_2) &= \frac{A_{1,2}}{4}\sin^2(\varphi_1-\varphi_2) \end{aligned}\right\} \quad \mathcal{V}_{AD}(\varphi_1;\varphi_2) = \frac{\cos^2(\varphi_1+\varphi_2) - \sin^2(\varphi_1-\varphi_2)}{\cos^2(\varphi_1+\varphi_2) + \sin^2(\varphi_1-\varphi_2)} \tag{S20}$$

In order to obtain the coincidences that we effectively see on the camera when a photon is recorded on the bucket detector, we have to integrate these expressions over the full φ_2 azimuthal angle, thus obtaining $C^{\alpha,\beta}_{(In)}(\mathbf{d}_{1,2};\varphi_1)$ and $C^{\alpha,\beta}_{(Out)}(\mathbf{d}_{1,2};\varphi_1)$, with $\mathbf{d}_{1,2} = (r_1, z_1; r_2, z_2)$. From these expressions we calculate the associated visibility distribution

$$\mathcal{V}(\varphi_1) = \frac{C^{\alpha,\beta}_{(Out)}(\mathbf{d}_{1,2};\varphi_1) - C^{\alpha,\beta}_{(In)}(\mathbf{d}_{1,2};\varphi_1)}{C^{\alpha,\beta}_{(Out)}(\mathbf{d}_{1,2};\varphi_1)} \tag{S21}$$

The spatial visibilities obtained for the 8 different polarisers configurations are

$$\begin{aligned} \mathcal{V}_{HH} &= 1 \\ \mathcal{V}_{HV} &= -1 \\ \mathcal{V}_{AH} &= \cos(2\varphi_1) \\ \mathcal{V}_{AV} &= -\cos(2\varphi_1) \\ \mathcal{V}_{HA} &= \mathcal{V}_{HD} = \mathcal{V}_{AA} = \mathcal{V}_{AD} = 0, \end{aligned} \tag{S22}$$

and coincide with those listed in Eq. (6) in the main text. The visibility spatial distributions for AH and AV are shown in Fig. 1.

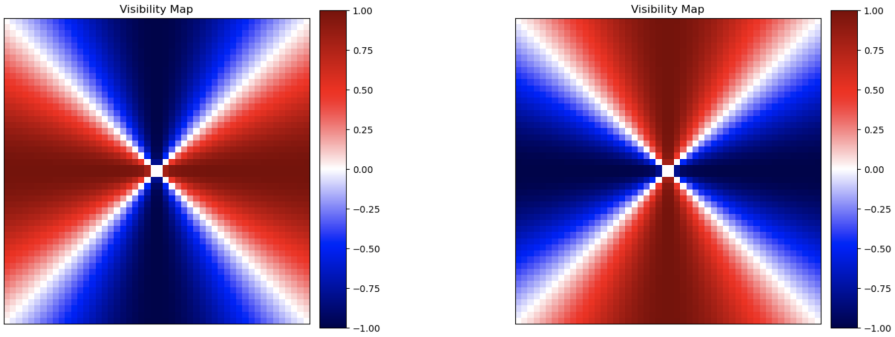

Supplementary Fig. 1. **Theoretical distributions of heralded spatially-varying coalescence.** Visibility distribution for A-H (left) and A-V (right) polarisers configurations.

2. RAW DATA

Background subtraction was applied to the measured coincidence counts to account for the erroneous contributions resulting from accidental detection and dark counts arising from both the APD and TimePix3 detectors. Supplementary Fig. 2 shows the data corresponding to Fig. 3 of the main text before background subtraction (as detailed in the Methods section). Visibilities for the raw HOM curves relating to the projection $(P_1, P_2) =$ (H,H) is $\mathcal{V} = 0.76 \pm 0.02$ and projection $(P_1, P_2) =$ (H,V) is $\mathcal{V} = -0.69 \pm 0.02$. The raw visibilities relating to all other projections are consistent with 0, accordant with the background subtracted results.

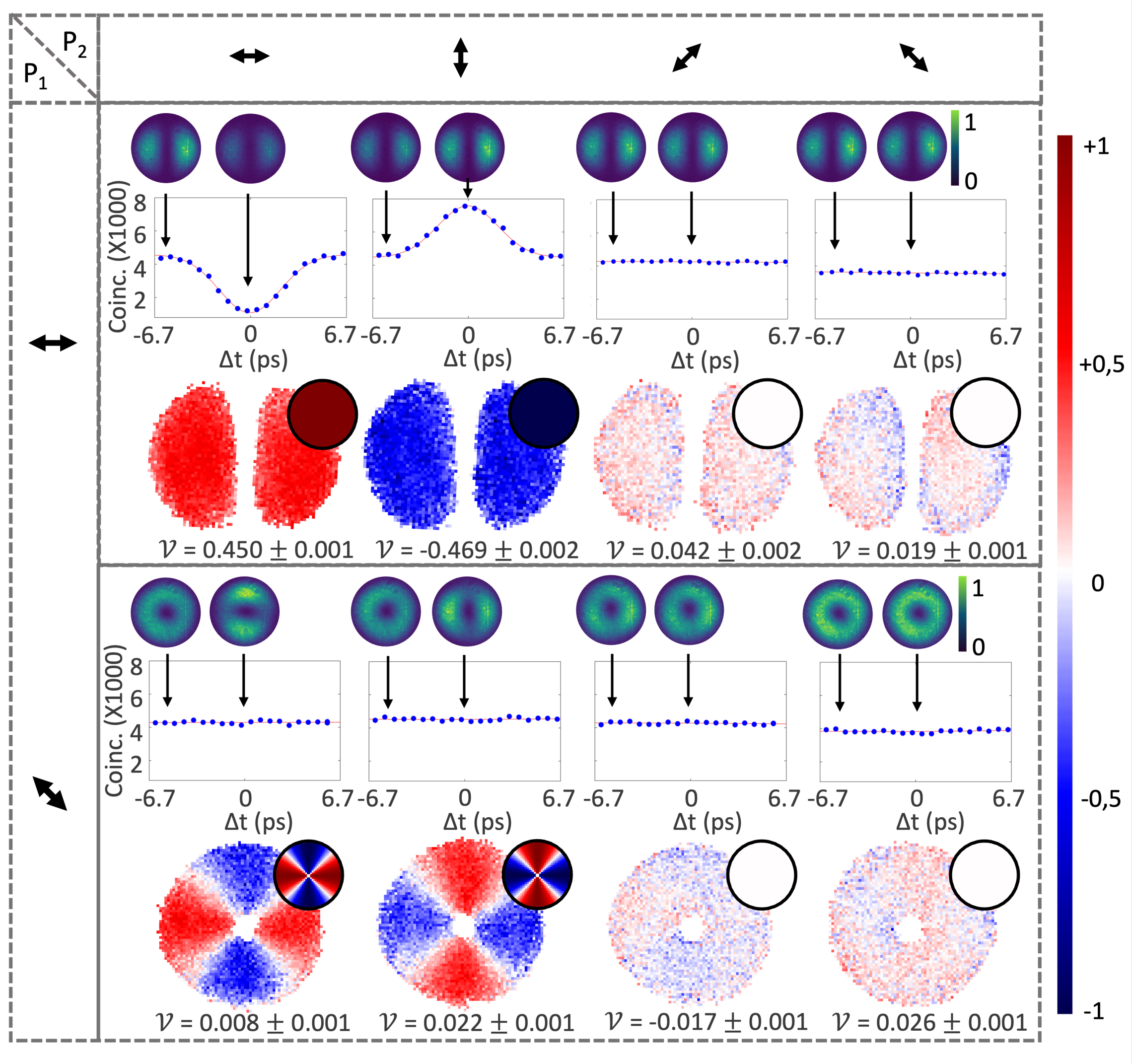

Supplementary Fig. 2. **Raw spatially tailored photon distributions.** Table of raw measured experimental outcomes (no background subtraction) for different choices of polarisation projections P1 (black arrows, row-wise) and P2 (black arrows, column-wise) as described in main text Fig. 3.

REFERENCES AND NOTES

1. C. K. Hong, Z. Y. Ou, L. Mandel Measurement of subpicosecond time intervals between two photons by interference. *Phys. Rev. Lett.* **59**, 2044–2046 (1987).

2. P. G. Kwiat, A. M. Steinberg, R. Y. Chiao Observation of a “quantum eraser”: A revival of coherence in a two-photon interference experiment. *Phys. Rev. A* **45**, 7729 (1992), 7739.

3. N. Harnchaiwat, F. Zhu, N. Westerberg, E. Gauger, J. Leach Tracking the polarisation state of light via Hong-Ou-Mandel interferometry. *Opt. Express* **28**, 2210–2220 (2020).

4. A. Khodadad Kashi, M. Kues Spectral Hong–Ou–Mandel interference between independently generated single photons for scalable frequency-domain quantum processing. *Laser Photon. Rev.* **15**, 2000464 (2021).

5. L. Hong, Y. Chen, L. Chen Delayed-choice quantum erasure with nonlocal temporal double-slit interference. *New J. Phys.* **25**, 053014 (2023).

6. T. Nitsche, S. de, S. Barkhofen, E. Meyer-Scott, J. Tiedau, J. Sperling, A. Gábris, I. Jex, C. Silberhorn Local versus global two-photon interference in quantum networks. *Phys. Rev. Lett.* **125**, 213604 (2020).

7. V. D’Ambrosio, G. Carvacho, I. Agresti, L. Marrucci, F. Sciarrino Tunable two-photon quantum interference of structured light. *Phys. Rev. Lett.* **122**, 013601 (2019).

8. S. Agne, T. Kauten, J. Jin, E. Meyer-Scott, J. Z. Salvail, D. R. Hamel, K. J. Resch, G. Weihs, T. Jennewein Observation of genuine three-photon interference. *Phys. Rev. Lett.* **118**, 153602 (2017).

9. A.J. Menssen, A. E. Jones, B. J. Metcalf, M. C. Tichy, S. Barz, W. S. Kolthammer, I. A. Walmsley Distinguishability and many-particle interference. *Phys. Rev. Lett.* **118**, 153603 (2017).

10. Y. Zhang, F. S. Roux, T. Konrad, M. Agnew, J. Leach, A. Forbes Engineering two-photon high-dimensional states through quantum interference. *Sci. Adv.* **2**, e1501165 (2016).

11. S.P. Walborn, A. N. de Oliveira, S. Pádua, C. H. Monken Multimode Hong-Ou-Mandel interference. *Phys. Rev. Lett.* **90**, 143601 (2003).

12. M. Hiekkamaki, R. Fickler, High-dimensional two-photon interference effects in spatial modes. *Phys. Rev. Lett.* **126**, 123601 (2021).

13. Z.F. Liu, C. Chen, J.M. Xu, Z.M. Cheng, Z.C. Ren, B.W. Dong, Y.C. Lou, Y.X. Yang, S.T. Xue, Z.H. Liu, W.Z. Zhu, X.L. Wang, H.T. Wang Hong-Ou-Mandel interference between two hyperentangled photons enables observation of symmetric and antisymmetric particle exchange phases. *Phys. Rev. Lett.* **129**, 263602 (2022).

14. A. Lyons, G. C. Knee, E. Bolduc, T. Roger, J. Leach, E. M. Gauger, D. Faccio Attosecond-resolution Hong-Ou-Mandel interferometry. *Sci. Adv.* **4**, eaap9416 (2018).

15. F. Triana-Arango, G. Ramos-Ortiz, R. Ramírez-Alarcón, Spectral considerations of entangled two-photon absorption effects in Hong–Ou–Mandel interference experiments. *J. Phys. Chem. A.* **127**, 2608–2617 (2023).

16. M. Jachura, R. Chrapkiewicz Shot-by-shot imaging of Hong-Ou-Mandel interference with an intensified scmos camera. *Opt. Lett.* **40**, 1540–1543 (2015).

17. R. Chrapkiewicz, M. Jachura, K. Banaszek, W. Wasilewski Hologram of a single photon. *Nat. Photon.* **10**, 576–579 (2016).

18. M. Lipka, M. Parniak Single-photon hologram of a zero-area pulse. *Phys. Rev. Lett.* **127**, 163601 (2021).

19. B. Ndagano, H. Defienne, D. Branford, Y. D. Shah, A. Lyons, N. Westerberg, E. M. Gauger, D. Faccio Quantum microscopy based on Hong–Ou–Mandel interference. *Nat. Photon.* **16**, 384–389 (2022).

20. F. Devaux, A. Mosset, P.A. Moreau, E. Lantz Imaging spatiotemporal Hong-Ou-Mandel interference of biphoton states of extremely high Schmidt number. *Phys. Rev. X* **10**, 031031 (2020).

21. J. Torgerson, D. Branning, C. H. Monken, L. Mandel, Violations of locality in polarization-correlation measurements with phase shifters. *Phys. Rev. A* **51**, 4400–4403 (1995).

22. J.-W. Pan, Z.-B. Chen, C.-Y. Lu, H. Weinfurter, A. Zeilinger, Marek Żukowski, Multiphoton entanglement and interferometry. *Rev. Mod. Phys.* **84**, 777–838 (2012).

23. M. Rezai, J. Wrachtrup, I. Gerhardt Coherence properties of molecular single photons for quantum networks. *Phys. Rev. X* **8**, 031026 (2018).

24. H. Rubinsztein-Dunlop, A. Forbes, M V Berry, M R Dennis, D. L. Andrews, M. Mansuripur, C. Denz, C. Alpmann, P. Banzer, T. Bauer, E. Karimi, L. Marrucci, M. Padgett, M. Ritsch-Marte, N. M. Litchinitser, N. P. Bigelow, C Rosales-Guzmán, A. Belmonte, J P Torres, T. W. Neely, M. Baker, R. Gordon, A. B. Stilgoe, J. Romero, A. G. White, R. Fickler, A. E. Willner, G. Xie, B. M. Morran, A. M. Weiner, Roadmap on structured light. *J. Opt.* **19**, 013001 (2017).

25. V. D'Ambrosio, N. Spagnolo, L. Del Re, S. Slussarenko, Y. Li, L. C. Kwek, L. Marrucci, S.P. Walborn, L. Aolita, F. Sciarrino, Photonic polarization gears for ultra-sensitive angular measurements. *Nat. Commun.* **4**, 2432 (2013).

26. R. Barboza, A. Babazadeh, L. Marrucci, F. Cardano, C. de Lisio, V. D’Ambrosio Ultra-sensitive measurement of transverse displacements with linear photonic gears. *Nat. Commun.* **13**, 1080 (2022).

27.G. H. Yuan, N. I. Zheludev Detecting nanometric displacements with optical ruler metrology. *Science* **364**, 771–775 (2019).

28. A. Bag, M. Neugebauer, P. Woźniak, G. Leuchs, P. Banzer Transverse kerker scattering for angstrom localization of nanoparticles. *Phys. Rev. Lett.* **121**, 193902 (2018).

29. N. Tischler, J. Stark, X. Zambrana-Puyalto, I. Fernandez-Corbaton, X. Vidal, G. Molina-Terriza, M. L. Juan All-optical self-referenced transverse position sensing with subnanometer precision. *ACS Photon.* **5**, 3628–3633 (2018).

30. C. Maurer, A. Jesacher, S. Fürhapter, S. Bernet, M. Ritsch-Marte Tailoring of arbitrary optical vector beams. *New J. Phys.* **9**, 78 (2007).

31. J. Wang, J.Y. Yang, I. M. Fazal, N. Ahmed, Y. Yan, H. Huang, Y. Ren, Y. Yue, S. Dolinar, M. Tur, A. E. Willner Terabit free-space data transmission employing orbital angular momentum multiplexing. *Nat. Photon.* **6**, 488–496 (2012).

32. I. Nape, B. Ndagano, A. Forbes Erasing the orbital angular momentum information of a photon. *Phys. Rev. A* **95**, 053859 (2017).

33. V. D'Ambrosio, I. Herbauts, E. Amselem, E. Nagali, M. Bourennane, F. Sciarrino, A. Cabello Experimental implementation of a Kochen-Specker set of quantum tests. *Phys. Rev. X* **3**, 011012 (2013).

34. M. Erhard, M. Krenn, A. Zeilinger Advances in high-dimensional quantum entanglement. *Nat. Rev. Phys.* **2**, 365–381 (2020).

35. S. Ecker, F. Bouchard, L. Bulla, F. Brandt, O. Kohout, F. Steinlechner, R. Fickler, M. Malik, Y. Guryanova, R. Ursin, M. Huber Overcoming noise in entanglement distribution. *Phys. Rev. X* **9**, 041042 (2019).

36. I. Nape, K. Singh, A. Klug, W. Buono, C. Rosales-Guzman, A. McWilliam, S. Franke-Arnold, A. Kritzinger, P. Forbes, A. Dudley, A. Forbes Revealing the invariance of vectorial structured light in complex media. *Nat. Photon.* **16**, 538–546 (2022).

37. V. D'Ambrosio, E. Nagali, S. P. Walborn, L. Aolita, S. Slussarenko, L. Marrucci, F. Sciarrino, Complete experimental toolbox for alignment-free quantum communication. *Nat. Commun.* **3**, 961 (2012).

38. P. Ornelas, I. Nape, R. de Mello Koch, A. Forbes, Non-local skyrmions as topologically resilient quantum entangled states of light. *Nat. Photon.* **18**, 258–266 (2024).

39. V. D'Ambrosio, G. Carvacho, F. Graffitti, C. Vitelli, B. Piccirillo, L. Marrucci, F. Sciarrino. Entangled vector vortex beams. *Phys. Rev. A* **94**, 030304 (2016).

40. X. Gao, Y. Zhang, A. D’Errico, A. Sit, K. Heshami, E. Karimi, Full spatial characterization of entangled structured photons. *Phys. Rev. Lett.* **132**, 063802 (2024).

41. F. Graffitti, V. D'Ambrosio, M. Proietti, J. Ho, B. Piccirillo, C. de Lisio, L. Marrucci, A. Fedrizzi Hyperentanglement in structured quantum light. *Phys. Rev. Res.* **2**, 043350 (2020).

42. K. Y. Bliokh, E. Karimi, M. J. Padgett, M. A. Alonso, M. R. Dennis, A. Dudley, A. Forbes, S. Zahedpour, S. W. Hancock, H. M. Milchberg, S. Rotter, F. Nori, Ş. K. Özdemir, N. Bender, H. Cao, P. B. Corkum, C. Hernández-García, H. Ren, Y. Kivshar, M. G. Silveirinha, N. Engheta, A. Rauschenbeutel, P. Schneeweiss, J. Volz, D. Leykam, D. A. Smirnova, K. Rong, B. Wang, E. Hasman, M. F. Picardi, A. V. Zayats, F. J. Rodríguez-Fortuño, C. Yang, J. Ren, A. B. Khanikaev, A. Alù, E. Brasselet, M. Shats, J. Verbeeck, P. Schattschneider, D. Sarenac, D. G. Cory, D. A. Pushin, M. Birk, A. Gorlach, I. Kaminer, F. Cardano, L. Marrucci, M. Krenn, F. Marquardt, Roadmap on structured waves. *J. Opt.* **25**, 103001 (2023).

43. T. Nagata, R. Okamoto, J. L. O'Brien, K. Sasaki, S. Takeuchi Beating the standard quantum limit with four-entangled photons. *Science* **316**, 726–729 (2007).

44. P. Walther, J.W. Pan, M. Aspelmeyer, R. Ursin, S. Gasparoni, A. Zeilinger De broglie wavelength of a non-local four-photon state. *Nature* **429**, 158–161 (2004).

45. S.-Y. Lee, H. Nha, Second-order superposition operations via Hong-Ou-Mandel interference. *Phys. Rev. A* **85**, 043816 (2012).

46. Y. Chen, S. Ecker, L. Chen, F. Steinlechner, M. Huber, R. Ursin Temporal distinguishability in Hong-Ou-Mandel interference for harnessing high-dimensional frequency entanglement *npj Quan. Inf.* **7**, 167 (2021).

47. D. Branning, W.P. Grice, R. Erdmann, I.A. Walmsley Engineering the indistinguishability and entanglement of two photons. *Phys. Rev. Lett.* **83**, 955–958 (1999).

48. D. Branning, W. Grice, R. Erdmann, I. A. Walmsley Interferometric technique for engineering indistinguishability and entanglement of photon pairs. *Phys. Rev. A* **62**, 013814 (2000).

49. N. Biagi, S. Francesconi, A. Zavatta, M. Bellini Photon-by-photon quantum light state engineering. *Progr. Quan. Electron.* **84**, 100414 (2022).

50. L. Marrucci, C. Manzo, D. Paparo Optical spin-to-orbital angular momentum conversion in inhomogeneous anisotropic media. *Phys. Rev. Lett.* **96**, 163905 (2006).

51. C. Cohen-Tannoudji, J. Dupont-Roc, G. Grynberg. *Photons and Atoms: Introduction to Quantum Electrodynamics* (John Wiley & Sons, New York, 1997).

52. G. Weihs, M. Reck, H. Weinfurter, A. Zeilinger Two-photon interference in optical fiber multiports. *Phys. Rev. A* **54**, 893–897 (1996).

53. C. Rosales-Guzmán, B. Ndagano, A. Forbes, A review of complex vector light fields and their applications. *J. Opt.* **20**, 123001 (2018).

54. X.-L. Wang, Y. Li, J. Chen, C.-S. Guo, J. Ding, H.-T. Wang, A new type of vector fields with hybrid states of polarization. *Opt. Express* **18**, 10786–10795 (2010).

55. Z. Liu, Y. Xie, W. Zhu, Q. Fu, F. Gao, G. Li, Y. Wang, X. Su, B. Zhang, S. Kumar Flexible method for generating arbitrary vector beams based on modified off-axis interference-type hologram encoding. *Photonics* **9**, 949 (2022).

56. H. P. Specht, J. Bochmann, M. Mücke, B. Weber, E. Figueroa, D. L. Moehring, G. Rempe Phase shaping of single-photon wave packets. *Nat. Photonics* **3**, 469–472 (2009).

57. B. Seron, L. Novo, N. J. Cerf Boson bunching is not maximized by indistinguishable particles. *Nat. Photonics* **17**, 702–709 (2023).

58. D. J. Brod, E. F. Galvão, A. Crespi, R. Osellame, N. Spagnolo, F. Sciarrino, Photonic implementation of boson sampling: A review. *Adv. Phot.* **1**, 034001 (2019).

59. G. Nirala, S. T. Pradyumna, A. Kumar, A. M. Marino Information encoding in the spatial correlations of entangled twin beams *Sci. Adv.* **9**, eadf9161 (2023).

60. M. Fisher-Levine, A. Nomerotski Timepixcam: A fast optical imager with time-stamping. *J. Instrum.* **11**, C03016 (2016).

61. C. Ianzano, P. Svihra, M. Flament, A. Hardy, G. Cui, A. Nomerotski, E. Figueroa Fast camera spatial characterization of photonic polarization entanglement. *Sci. Rep.* **10**, 6181 (2020).

62. V. Vidyapin, Y. Zhang, D. England, B. Sussman Characterisation of a single photon event camera for quantum imaging. *Sci. Rep.* **13**, 1009 (2023).

63. J.-Å. Larsson, Loopholes in bell inequality tests of local realism. *J. Phys. A Math. Theor*. **47**, 424003 (2014).

64. B. Courme, C. Vernière, P. Svihra, S. Gigan, A. Nomerotski, H. Defienne Quantifying high-dimensional spatial entanglement with a single-photon-sensitive time-stamping camera. *Opt. Lett.* **48**, 3439–3442 (2023).

65. Edgar, M. P., Tasca D.S., Izdebski F., Warburton R.E., Leach J., Agnew M., Buller G.S., Boyd R.W., Padgett M.J. Imaging high-dimensional spatial entanglement with a camera. *Nat. Commun.* **3**, 984 (2012).

66. P.-A. Moreau, J. Mougin-Sisini, F. Devaux, E. Lantz, Realization of the purely spatial Einstein-Podolsky-Rosen paradox in full-field images of spontaneous parametric down-conversion. *Phys. Rev. A* **86**, 010101 (2012).

67. P.-A. Moreau, F. Devaux, E. Lantz, Einstein-Podolsky-Rosen paradox in twin images. *Phys. Rev. Lett.* **113**, 160401 (2014).

68. M. Reichert, H. Defienne, J. W. Fleischer Massively parallel coincidence counting of high-dimensional entangled states. *Sci. Rep.* **8**, 7925 (2018).

69. M. Dąbrowski, M. Parniak, W. Wasilewski, Einstein–Podolsky–Rosen paradox in a hybrid bipartite system. *Optica* **4**, 272–275 (2017).

70. M. Dąbrowski, M. Mazelanik, M. Parniak, A. Leszczyński, M. Lipka, W. Wasilewski, Certification of high-dimensional entanglement and Einstein-Podolsky-Rosen steering with cold atomic quantum memory. *Phys. Rev. A* **98**, 042126 (2018).

71. B. Ndagano, H. Defienne, A. Lyons, I. Starshynov, F. Villa, S. Tisa, D. Faccio Imaging and certifying high-dimensional entanglement with a single-photon avalanche diode camera *npj Quantum Inf.* **6**, 94 (2020).

72. B. Eckmann, B. Bessire, M. Unternährer, L. Gasparini, M. Perenzoni, A. Stefanov Characterization of space-momentum entangled photons with a time resolving cmos spad array. *Opt. Express* **28**, 31553–31571 (2020).

73. C. Cohen-Tannoudji, J. Dupont-Roc, G. Grynberg, *Photons and Atoms: Basic Processes and Applications* (John Wiley & Sons, New York, 1992).
